# Aseptically Filled Tofu (Microorganisms and Viruses): Summary

**DOI:** 10.14252/foodsafetyfscj.D-19-00018

**Published:** 2019-12-27

**Authors:** Food Safety Commission of Japan

## Abstract

Aseptically filled Tofu was necessary to be refrigerated under the previous standards for tofu. Food Safety Commission of Japan (FSCJ) was requested by the Ministry of Health, Labour and Welfare (MHLW) to assess a food safety risk to human health on changing the standards to ambient temperature storage. *Clostridium botulinum* and *Bacillus cereus* can be significant hazards. Assuming that manufacturing procedures including sterilizing processes follow the MHLW-indicated condition, under a full hygienic control, *C. botulinum* and *B. cereus* would not exist in the final products. Therefore, FSCJ concludes that there are no substantial risks of human health irrespective of the change in the storage standard for aseptically filled Tofu.

## Conclusion in Brief

Aseptically filled Tofu was necessary to be refrigerated under the previous standards for tofu. Food Safety Commission of Japan (FSCJ) was requested by the Ministry of Health, Labour and Welfare (MHLW) to assess a food safety risk to human health on changing the standards to ambient temperature storage.

Aseptically filled Tofu, produced under the procedure specified by MHLW, is expectedly kept and distributed on markets for prolonged periods at ambient temperature.

If *Clostridium botulinum* and *Bacillus cereus*, which were corresponding to significant hazards, would remain in the final products, these may have a concern to yield an adverse effect on human health.

Assuming that manufacturing procedures including sterilizing processes follow the MHLW-indicated condition, under a full hygienic control based on “Guidelines on Management and Operation Standards to be Observed by Food-Related Business Operators (Notice Shoku-an No. 1014-1 of October 14, 2014)”, *C. botulinum* and *B. cereus* would not exist in the final products.

Therefore, FSCJ concludes that there are no substantial risks of human health irrespective of the change in the storage standard for aseptically filled Tofu.

A soybean soaking process must be under an appropriate control of the process to prevent a growth of the bacteria to a number required for producing a highly heat stable toxin.

In addition, a monitoring system to secure a condition at 120°C for 4 min or the more severe is necessary for the process control, and the corrective action must be taken immediately at inappropriate conditions.

Packages of aseptically filled Tofu are required to be resistant to physical stimuli and to be capable to prevent a damage that may cause contamination with bacteria. Moreover, indications to consumers are necessary to provide clearly the relevant information: 1) To keep refrigerated for conventional Tofu products; and 2) To be allowed to keep in ambient temperature for aseptically filled Tofu.

